# Transcriptomic profiling of mare endometrium at different stages of endometrosis

**DOI:** 10.1038/s41598-023-43359-5

**Published:** 2023-09-27

**Authors:** A. Szóstek-Mioduchowska, A. Wójtowicz, A. Sadowska, B. Moza Jalali, M. Słyszewska, K. Łukasik, A. Gurgul, T. Szmatoła, M. Bugno-Poniewierska, G. Ferreira-Dias, D. J. Skarzynski

**Affiliations:** 1 Department of Reproductive Immunology and Pathology, Institute of Animal Reproduction and Food Research Polish Academy of Sciences in Olsztyn, Olsztyn, Poland; 2 Center for Experimental and Innovative Medicine, University of Agriculture in Cracow, Cracow, Poland; 3 Department of Animal Reproduction, Anatomy and Genomics, University of Agriculture in Cracow, Cracow, Poland; 4 Centre for Interdisciplinary Research in Animal Health, Faculty of Veterinary Medicine, University of Lisbon, Lisbon, Portugal

**Keywords:** Molecular biology, Reproductive biology

## Abstract

In the current study, transcriptome profiles of mare *endometrium*, classified into categories I, IIA, and IIB according to Kenney and Doig, were compared using RNA sequencing, analyzed, and functionally annotated using in silico analysis. In the mild stage (IIA) of endometrosis compared to category I *endometrium*, differentially expressed genes (DEGs) were annotated to inflammation, abnormal metabolism, wound healing, and quantity of connective tissue. In the moderate stage (IIB) of endometrosis compared to category I *endometrium*, DEGs were annotated to inflammation, fibrosis, cellular homeostasis, mitochondrial dysfunction, and pregnancy disorders. Ingenuity pathway analysis (IPA) identified cytokines such as transforming growth factor (TGF)-β1, interleukin (IL)-4, IL-13, and IL-17 as upstream regulators of DEGs associated with cellular homeostasis, metabolism, and fibrosis signaling pathways. In vitro studies showed the effect of these cytokines on DEGs such as *ADAMTS1*, *-4*, *-5*, *-9*, and *HK2* in endometrial fibroblasts at different stages of endometrosis. The effect of cytokines on ADAMTS members’ gene transcription in fibroblasts differs according to the severity of endometrosis. The identified transcriptomic changes associated with endometrosis suggest that inflammation and metabolic changes are features of mild and moderate stages of endometrosis. The changes of *ADAMTS-1*, *-4*, *-5*, *-9*, in fibrotic *endometrium* as well as in endometrial fibroblast in response to TGF-β1, IL-4, IL-13, and IL-17 suggest the important role of these factors in the development of endometrosis.

## Introduction

Equine endometrosis is a degenerative chronic condition of the uterus, defined as fibrosis that develops around the endometrial glands and in the stroma^1–3^. Endometrial fibrosis in mares is associated with excessive deposition of extracellular matrix (ECM) components and pathological changes in the endometrial glands, such as cystic dilation and atrophy or hypertrophy of the epithelium^1–3^. This condition affects  the *endometrium* structure as well as the uterine microenvironment and leads to early pregnancy loss^3, 4^. Depending on the degree of structural changes, the *endometria* are divided into four categories: I (without fibrosis), and IIA, IIB, III which correspond to mild, moderate, and severe fibrosis, inflammatory infiltrates, and degree of dilation of both endometrial glands and lymphatic vessels, respectively^2^. In endometrosis, the expected foaling rate is 80–90%, 50–80%, 10–50%, and 10% for categories I, IIA, IIB, and III *endometria*, respectively^2^. Therefore, endometrosis is a serious problem in the horse-breeding industry.

The mechanisms underlying tissue fibrosis are very complex with several overlapping stages including inflammation, proliferation, and remodeling. It is worth emphasizing that fibrosis is not only limited to mare *endometrium*. According to Zeisberg and Kalluri^5^, chronic loss of function of most organs, including the heart, intestine, kidney, liver, lung, bone marrow, and skin is associated with fibrosis, contributing to an estimated one-third of premature deaths worldwide. Fibrosis across the organs shows many histomorphological similarities and shares pathways and mechanisms contributing to its development^5^. Despite many similarities, there are organ-specific mechanisms responsible for fibrosis development.

In mare endometrosis, research has mainly focused on the changes in the endometrial expression of inflammatory markers, and components of ECM including synthases of prostaglandin, cytokines, and metalloproteinases (MMP) at the different stages of endometrosis^3, 6–11^. Our previous results described the effects of mediators of inflammation on cell proliferation, fibroblast to myofibroblast differentiation, and the expression of MMPs and their tissue inhibitors (TIMPs)^10–13^. Recently, transcriptomic analysis of mare *endometrium* with severe endometrosis in anestrus was examined^14^. In endometrium with severe fibrotic changes, the expression of genes was annotated to processes such as to collagen catabolic process, lipid metabolic process, one-carbon metabolic process, defense response to the virus, metalloendopeptidase activity, and extracellular matrix organization. However, to the best of our knowledge, the transcriptomic analysis of endometrial tissue in categories IIA and IIB of endometrosis in the estrous cycle was not described so far.

The application of transcriptomic analysis allows for filling gaps in understanding the nature of endometrial fibrosis as well as identifying the novel pathways and regulators that participate in the development of endometrosis. Therefore, we compared the transcriptome profiles of mare *endometrium* at different stages of endometrosis using RNA sequencing (RNA-seq). Furthermore, in in vitro study, the effect of transforming growth factor (TGF)-β1 and interleukins (IL)-4, -13, -17 on the expression of genes selected based on global transcriptomic analysis was investigated in endometrial fibroblasts.

## Results

### Experiment 1. Transcriptomic analysis of endometrial tissue at different stages of endometrosis at the follicular phase of the estrous cycle

In this experiment, we generated RNA-Seq data for 9 pools of 27 samples belonging to all three studied groups. After rejecting low-quality reads, we used from 14.6 to 20.1 M high-quality reads per sample (17.7 M on average). Of the reads, 89.06% on average were successfully mapped against the reference genome (Supplementary Table 1).

#### Differential changes in endometrial transcriptome at different stages of endometrosis

A comparison of the transcriptomes of *endometria* collected from categories IIA and IIB with that of category I revealed significant differences in gene expression between healthy and fibrotic *endometrium*. Most changes were observed in the category IIB *endometrium* with moderate changes due to endometrial fibrosis. We observed significant differences in the expression of 230 (58 up-regulated and 172 down-regulated), and 1101 (598 up-regulated and 503 down-regulated) in categories IIA and IIB compared to category I *endometria*, respectively. Additionally, significant differences in the expression of 2111 (1152 up-regulated and 959 down-regulated) transcripts in categories IIB compared to category IIA *endometria* were observed. In each comparison, besides known genes, several novel transcripts were identified as differentially expressed. A complete list of differentially expressed transcripts in endometrial tissue in categories IIA and IIB vs I as well as IIB vs IIA is reported in Supplementary Table 2.

#### Comparison of changes in endometrial transcriptome at different stages of endometrosis

**Figure 1:**
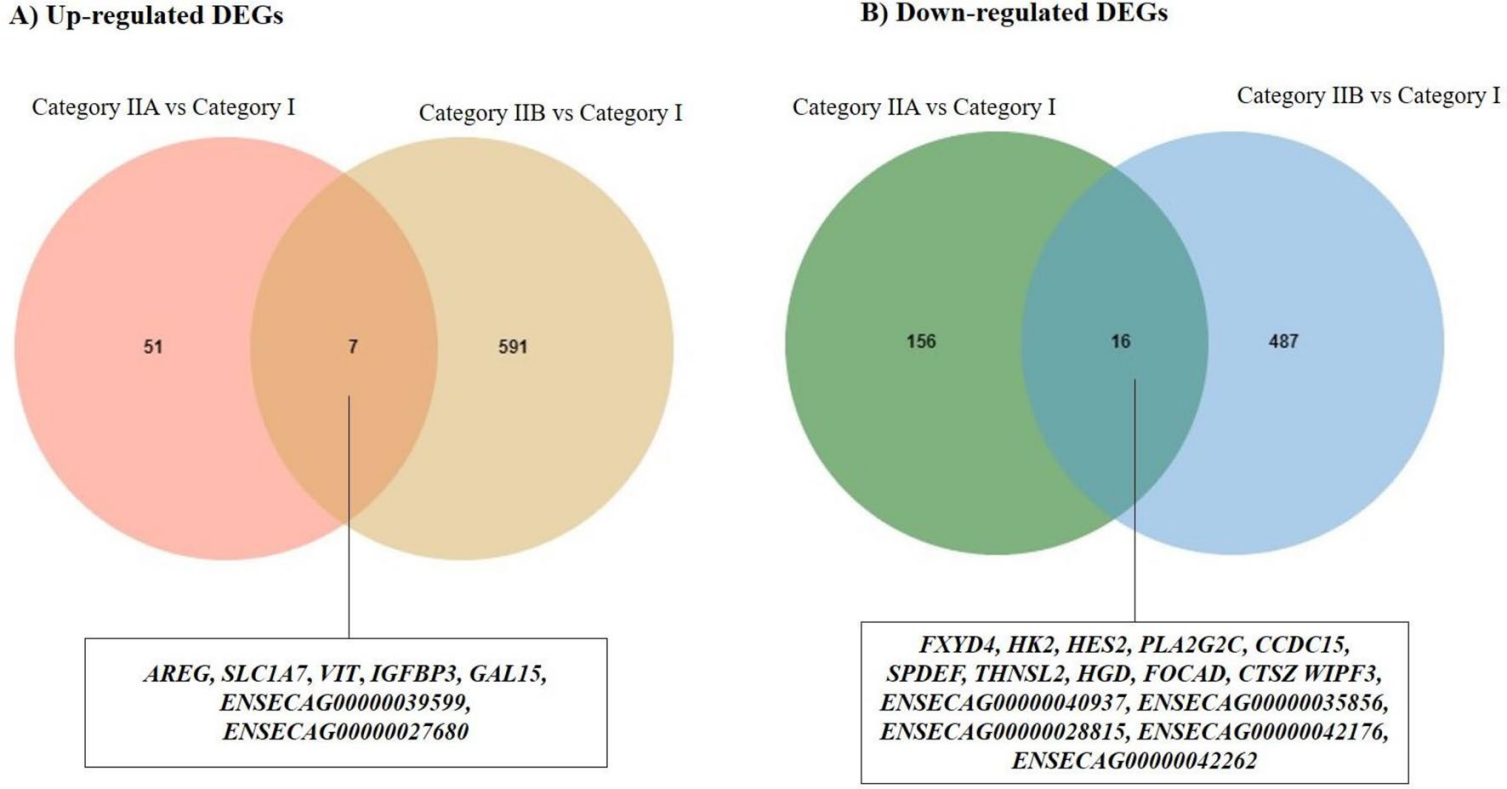
Shared and unique DEGs in* endometria* at different stages of endometrosis. Venn diagram showing up-regulated (**A**) and down-regulated (**B**) shared and unique DEGs in categories IIA and IIB in comparison to category I *endometria* in the follicular phase of the estrous cycle.

The lists of up-and down-regulated DEGs across the categories IIA vs I and IIB vs I *endometria* were uploaded to jvenn software (http://jvenn.toulouse.inra.fr/app/example.html) to visualize genes whose expression changed across more than one endometrosis categories or only in a specific category as compared to the healthy *endometrium* (Fig. 1A and B). Change in 23 genes was common to categories IIA vs I and IIB vs I *endometria* of which seven were up-regulated and 16 were down-regulated (Fig. 1A and B, listed in Supplementary Table 2).

#### Analysis of biological processes, pathways, and upstream regulators of identified DEGs

##### Biological processes

To understand the functional significance of gene expression changes in endometrial tissue in categories IIA vs I, IIB vs I, as well as IIB vs IIA core analysis available in the IPA was used to identify pathways that were significantly enriched by the DEGs. Important enriched pathways and functional categories relevant to the process of endometrosis along with their associated DEGs in categories IIA vs I, IIB vs I, and IIB vs IIA *endometria* are presented in Table 1. A complete list of comparison analyses, comparing disease and biological functions, canonical pathways, and upstream regulators in categories IIA vs I, IIB vs I as well as IIB vs IIA *endometria* are presented in Supplementary Table 3.

**Table 1:** IPA analysis overview: selected molecular and cellular function, and canonical pathways associated with differentially expressed genes between categories: IIA vs I *endometria*, IIB vs I *endometria*, IIB vs IIA *endometria*.

Comparison	Molecular and cellular functions	*p*-value	No. of genes	Canonical pathways	− log (p-value)	No. of genes
IIA vs I	Inflammation of organ	2.59E-08	47	NRF2-mediated oxidative stress response	3.13	8
	Quantity of connective tissue	8.89E-06	22	Ferroptosis signaling pathway	3.13	6
	Synthesis of fatty acid	7.64E-06	15	Inhibition of matrix metalloproteases	2.41	3
	Abnormal metabolism	1.37E-05	16	Wound Healing signaling pathway	2.33	7
	Metabolism of reactive oxygen species	2.92E-05	20	HIF1α signaling pathway	2.14	6
	Cellular infiltration by macrophages	0.000683	9	IL-13 signaling pathway	1.83	4
	Metabolism of peptide	0.000342	11	IL-17 signaling	1.73	5
	Metabolism of carbohydrate	0.000128	21	Pulmonary fibrosis idiopathic signaling pathway	1.3	6
IIB vs I	Necrosis	4.55E-16	284	Gαq signaling	6.12	22
	Angiogenesis	1.09E-12	127	Mitochondrial dysfunction	4.42	19
	Apoptosis	4.54E-11	255	Pulmonary fibrosis idiopathic signaling pathway	4.11	29
	Disorder of pregnancy	8.78E-10	99	Oxidative phosphorylation	4.00	14
	Growth of connective tissue	6.45E-10	82	Endotelin-1 signaling	3.74	19
	Cellular homeostasis	8.34E-09	170	HIF1α signaling pathway	3.3	19
	Synthesis of lipid	6.07E-07	85	Inhibition of matrix metalloproteases	3.18	7
	Inflammation of organ	3.66E-07	145	IL-13 signaling pathway	2.26	11
	Cell proliferation of fibroblasts	7.67E-06	99	WNT/β-catenin signaling	1.41	12
	Fibrosis	2.26E-06	84	Th1 and Th2 activation pathway	1.14	11
IIB vs IIA	Cell movement of leukocytes	6.6E-19	197	Neutrophil extracellular trap signaling pathway	5.11	29
	Apoptosis	2.11E-19	492	Inhibition of matrix metalloproteases	3.38	10
	Differentiation of connective tissue cells	4.26E-16	152	Mitochondrial dysfunction	3.27	43
	Fibrosis	1.5E-16	205	Pulmonary fibrosis idiopathic signaling pathway	3.22	41
	Disorder of pregnancy	1.44E-15	188	Th2 pathway	2.95	21
	Transport of molecules	2.62E-12	286	Autophagy	2.02	26
	Synthesis of lipid	1.91E-12	164	Oxidative phosphorylation	1.78	15

**Figure 2:**
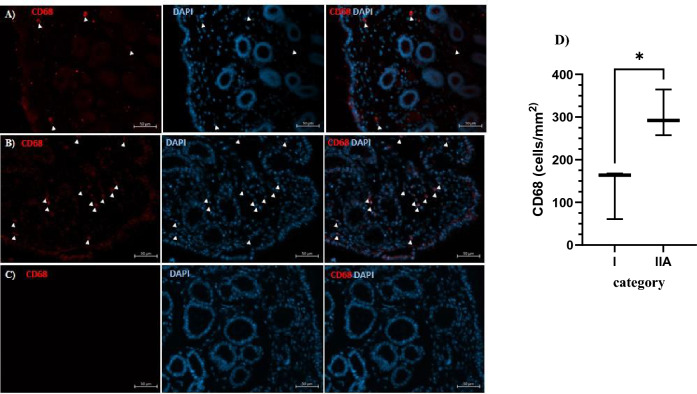
Endometrial infiltration of macrophages. Representative immunostaining of CD68 in categories I (**A**) and IIA (**B**) *endometria* in the follicular phase of the estrous cycle. (**C**) negative control; scale bar, 50 µM. The white arrows indicate the macrophages. (**D**) The number of positive CD68^+^ cells counted in endometrial regions; Asterisks denote statistical differences (*P < 0.05).

The functional annotation of DEGs identified in category IIA vs I *endometria* was associated with functions such as inflammation of the organ, organismal death, the quantity of interleukin, cellular infiltration by macrophages, leukocyte migration, cell movement, the quantity of macrophages, abnormal metabolism. Infiltration of macrophages in *endometria* was confirmed by immunofluorescence staining where a significantly higher number of macrophages was observed in category IIA *endometrium* (Fig. 2A–D). Additionally, in category IIA vs I *endometria*, DEGs led to the enrichment of canonical pathways that can be linked to pathological processes such as fibrosis, ferroptosis signaling pathway, inhibition of matrix metalloproteases, wound healing signaling pathway, HIF-1α signaling pathway, IL-13 pathway, and IL-17 signaling.

In category IIB vs I *endometria*, many functional categories and pathways that can lead to the pathology of fibrosis were enriched. The DEGs in this category were annotated to processes such as leukocyte migration, organization of cytoskeleton, apoptosis, organismal injury and abnormalities, growth of connective tissue, cellular homeostasis, and proliferation of fibroblasts and epithelial cells.

Canonical pathways that were enriched among DEGs associated with category IIB vs I *endometria* included mitochondrial dysfunction, pulmonary fibrosis idiopathic signaling pathway, oxidative phosphorylation, endothelin-1 signaling, HIF-1α signaling pathway, IL-13 pathway, inhibition of matrix metalloproteases, Th1 and Th2 activation pathway.

Furthermore, in category IIB vs IIA *endometria*, DEGs were annotated to decrease the activation of processes such as recruitment of phagocytes, transport of molecules, organismal death. Canonical pathways that were enriched among DEGs associated with category IIB vs IIA *endometria* included inhibition of matrix metalloproteases, mitochondrial dysfunction, pulmonary fibrosis idiopathic signaling pathway, epithelial adherent junction signaling, production of nitric oxide and reactive oxygen species in macrophages, DNA methylation and transcriptional repression signaling, phagosome formation, oxidative phosphorylation, and mitochondrial dysfunction.

**Figure 3:**
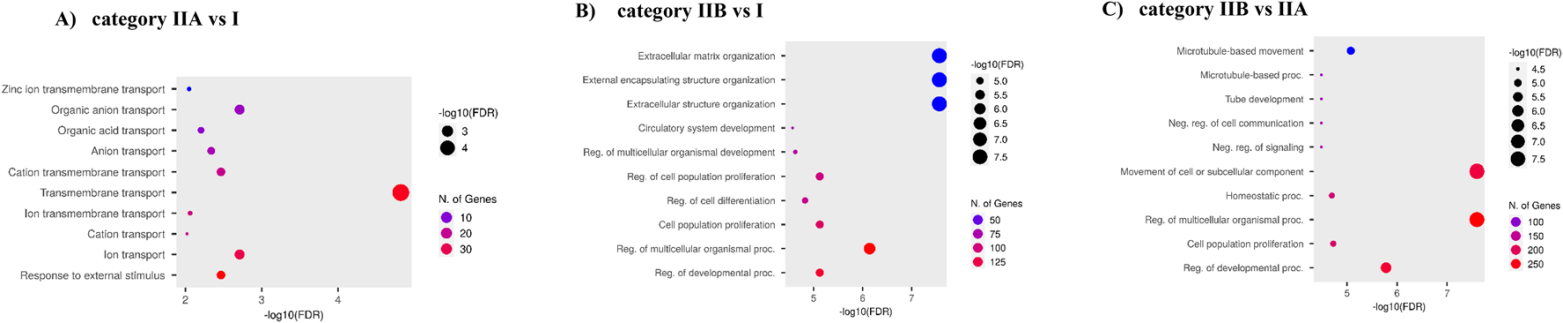
Top 10 significant GO biological processes in *endometria* at different stages of endometrosis. (**A**) category IIA vs I *endometria*, (**B**) category IIB vs I *endometria*, (**C**) category IIB vs IIA *endometria* visualized using ShinyGO software (*FDR* ≤ 0.05; hypergeometric test followed by FDR correction).

GO biological processes were analyzed using ShinyGO version 0.741 (*FDR* ≤ 0.05; hypergeometric test followed by FDR correction)^15^, and the top—10 GO terms or related biological processes in categories IIA vs I, IIB vs I, IIB vs IIA *endometria* are shown in Fig. 3.

##### Upstream regulators

The upstream regulator analysis tool in IPA was used to predict the potential upstream regulators of DEGs in categories IIA and IIB vs I *endometria*. Several factors including cytokines that are known to be associated with fibrosis were predicated to be activated or inhibited. A complete list of upstream regulators in categories IIA and IIB vs I as well as IIB vs IIA *endometria* are presented in Supplementary Table 3. The selected upstream regulators including cytokines and growth factors associated with DEGs between categories: IIA and IIB vs I as well as IIB vs IIA *endometria* are presented in Table 2. In category IIA vs I *endometria*, FLCN, CEBPA, GLI1, miR-16-5p, LAMA4, KLF3, MMP9, lipoteichoic acid, LDL, and FGF2 were shown as activated upstream regulators, (Table 2, Supplementary Table 3). In category IIB vs I *endometria*, growth factors such as BMP10, LEP, IGF2, and cytokines including IL-1β, LIF, IL-1α, TNFSF13B, C5, TNF, CXCL12, CD40LG, IL-33, EPO, IL-6, IL-15, TIMP1 were shown as activated upstream regulators (Table 2, Supplementary Table 3). In category IIB vs IIA *endometria*, collagenase, TNF, STAT3, IL-13, PDGD, LH was shown as activated upstream regulators, (Table 2, Supplementary Table 3).

**Table 2:** IPA analysis overview: selected upstream regulators associated with differentially expressed genes between categories: IIA vs I, IIB vs I, III vs I *endometria*.

Group	Upstream regulator	Molecule type	*p*-value	Target molecules in dataset
IIA vs I	IL-6	Cytokine	2.15E-06	22
	IL-1β	Cytokine	1.9E-06	28
	FGF	Growth factor	1.72E-06	17
	TGF-β1	Growth factor	1.2E-06	39
	IL-4	Cytokine	5.34E-05	25
	Gli1	Transcription regulator	0.000214	14
	AREG	Growth factor	0.00696	4
	HIF-1α	Transcription regulator	0.0238	10
IIB vs I	TNF	Cytokine	4.29E-14	151
	TGF-β1	Growth factor	2.34E-13	150
	IL-1β	Cytokine	3.59E-09	35
	LIF	Cytokine	8.62E-09	30
	IL6	Cytokine	1.79E-08	69
	IL-13	Cytokine	0.000717	37
	IL-17A	Cytokine	0.00414	23
	BMP10	Growth factor	0.00137	11
IIB vs IIA	TNF	Cytokine	3.55E-26	16
	STAT3	Transcription regulator	1.32E-15	16
	IL-13	Cytokine	1.16E-09	16
	PDGF	Growth factor	0.000214	17
	Collagenase	Enzyme	0.00586	13

#### qPCR validation of transcriptome sequencing

For qPCR validation of transcriptome sequencing results, 4 DEGs (*HK2*, *CXCR4*, *ADAMTS9, SLC25A29*) were investigated. A comparison of log2change and p-value of DEGs obtained after qPCR and data analysis of RNA-seq is presented in Supplementary Table 4. The qPCR analysis showed the direction of changes in the expression of all the evaluated DEGs.

### Experiment 2. The effect of cytokines on the differentially expressed genes in endometrial fibroblasts

#### Transforming growth factor-β1

**Figure 4:**
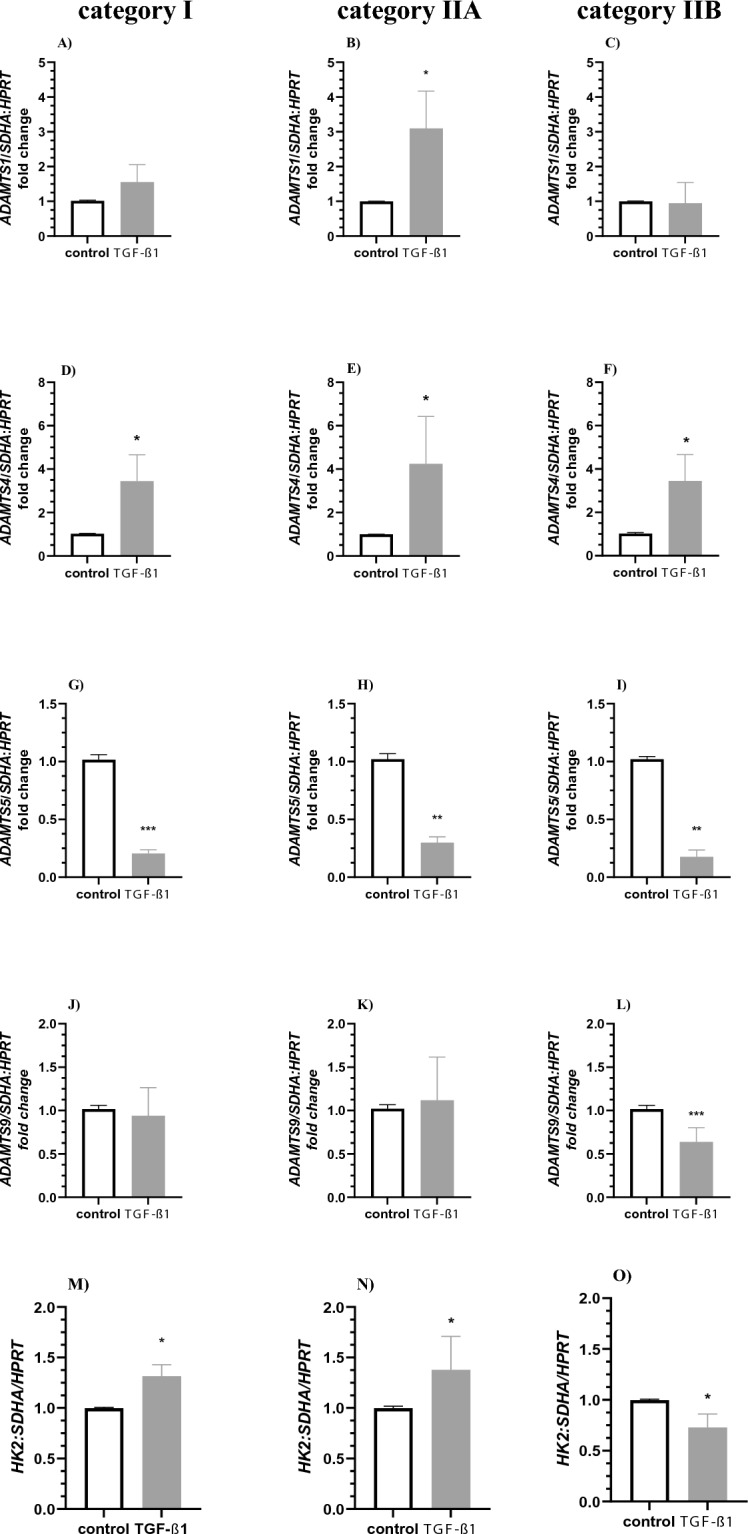
The effect of TGF-β1 on expression of *ADAMTS* and *HK2* mRNA expression in equine endometrial fibroblasts. TGF-β1 treatment (10 ng/ml) on (**A**–**C**) *ADAMTS1*, (**D**–**F**) *ADAMTS4*, (**G**–**I**) *ADAMTS5*, (**J**–**L**) *ADAMTS9*, (**M**–**O**) *HK2* mRNA transcription in in vitro cultured fibroblast derived from categories I, IIA and IIB *endometria*. Results are presented as a fold change. Asterisks denote statistical differences (*P < 0.05; **P < 0.01; ***P < 0.001) as determined by the nonparametric Mann–Whitney *U* test.

Transforming growth factor-β1 treatment did not affect the expression of *ADAMTS1* in fibroblasts derived from categories I and IIB (Fig. 4A and C; P > 0.05), but it increased the expression of *ADAMTS1* in fibroblasts derived from category IIA *endometrium* (Fig. 4B; P < 0.05). Additionally, TGF-β1 treatment induced an increase in *ADAMTS4* in fibroblasts derived from categories I, IIA, and IIB *endometria* (Fig. 4D–F; P < 0.05). In contrast, treatment with TGF-β1 caused a decrease in *ADAMTS5* mRNA transcription in the fibroblasts derived from categories I (Fig. 4G; P < 0.001), IIA and IIB *endometria* (Fig. 4H–I; P < 0.01). Transforming growth factor-β1 treatment did not affect the expression of *ADAMTS9* in fibroblasts derived from categories I and IIA (Fig. 4J and K; P > 0.05) but it decreased the expression of *ADAMTS9* in the fibroblasts derived from category III *endometrium* (Fig. 4L; P < 0.001).

Transforming growth factor-β1 treatment increased *HK2* mRNA transcription in cultured fibroblasts derived from category I (Fig. 4M; P < 0.05) and category IIA (Fig. 4N; P < 0.05), but decreased *HK2* mRNA transcription in cultured fibroblasts derived from category IIB * endometria *(Fig. 4O; P < 0.05).

### IL-4, IL-13, and IL-17

**Figure 5:**
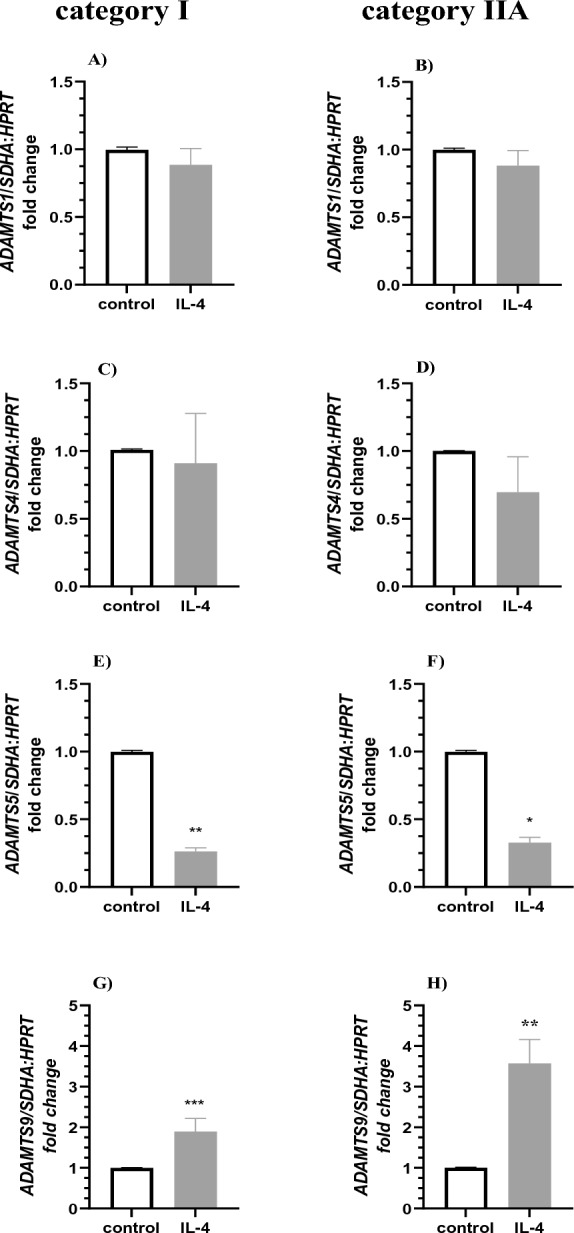
The effect of IL-4 on expression of *ADAMTS* mRNA expression in equine endometrial fibroblasts. IL-4 treatment (10 ng/ml) on (**A**,**B**) *ADAMTS1*, (**C**,**D**) *ADAMTS4*, (**E**,**F**) *ADAMTS5*, (**G**,**H**) *ADAMTS9* mRNA transcription in in vitro cultured fibroblast derived from (**A**,**C**,**E**,**G**) categories I and (**B**,**D**,**F**,**H**) IIA *endometria*. Results are presented as a fold change. Asterisks denote statistical differences (*P < 0.05; **P < 0.01) as determined by the nonparametric Mann–Whitney *U* test.

Interleukin 4 treatment did not affect the expression of *ADAMTS1* and *ADAMTS4* in fibroblasts derived from categories I and IIA *endometria* (Fig. 5A–D; P > 0.05). However, IL-4 treatment decreased the expression of *ADAMTS5* (Fig. 5E,F; P < 0.01, P < 0.05) and increased the expression of *ADAMTS9* in cultured fibroblasts derived from categories I and IIA *endometria* (Fig. 5G,H; P < 0.001; P < 0.05).

**Figure 6:**
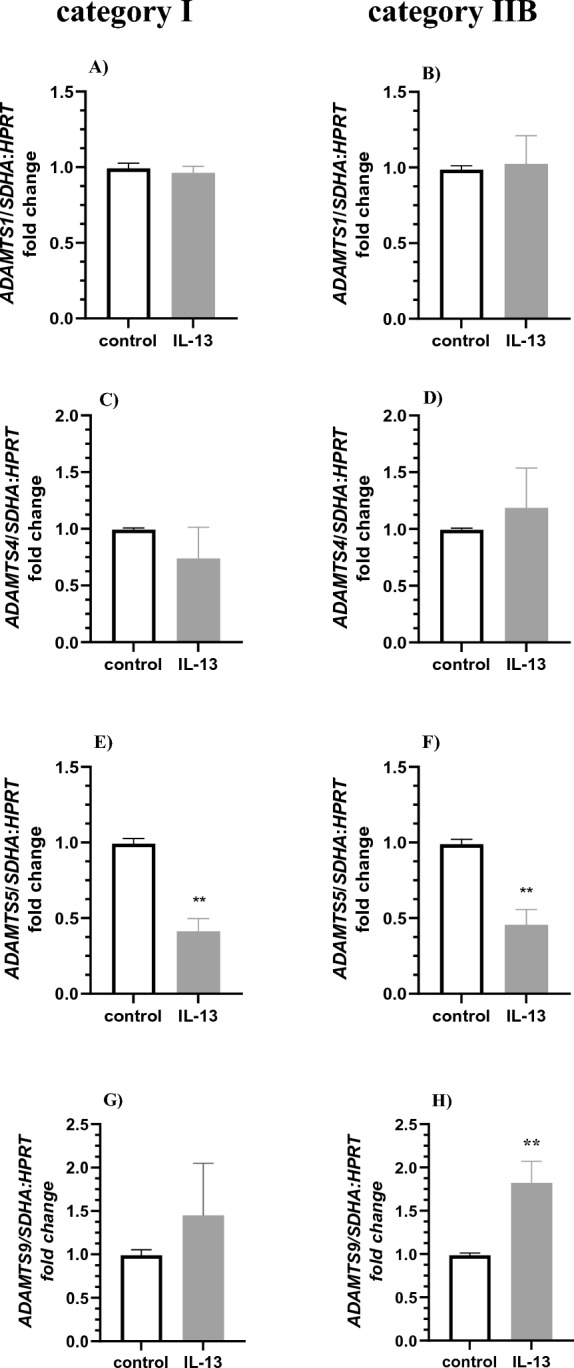
The effect of IL-13 on expression of *ADAMTS* mRNA expression in equine endometrial fibroblasts. IL-13 treatment (10 ng/ml) on (**A**,**B**) *ADAMTS1*, (**C**,**D**) *ADAMTS4*, (**E**,**F**) *ADAMTS5*, (**G**,**H**) *ADAMTS9* mRNA transcription in in vitro cultured fibroblast derived from (**A**,**C**,**E**,**G**) categories I and (**B**,**D**,**F**,**H**) IIB *endometria*. Results are presented as a fold change. Asterisks denote statistical differences (**P < 0.01) as determined by the nonparametric Mann–Whitney *U* test.

Interleukin 13 treatment did not affect the expression of *ADAMTS1* and *ADAMTS4* in fibroblasts derived from categories I and IIB (Fig. 6A–D, P > 0.05) but decreased *ADAMTS5* mRNA transcription in cultured fibroblasts derived from category I and IIB *endometria* (Fig. 6E–F; P < 0.01). In turn, IL-13 treatment did not affect the expression of *ADAMTS9* in fibroblasts derived from category I (Fig. 6G; P < 0.05) but increased the expression of *ADAMTS9* in fibroblasts derived from category IIB *endometrium* (Fig. 6H; P < 0.01).

**Figure 7:**
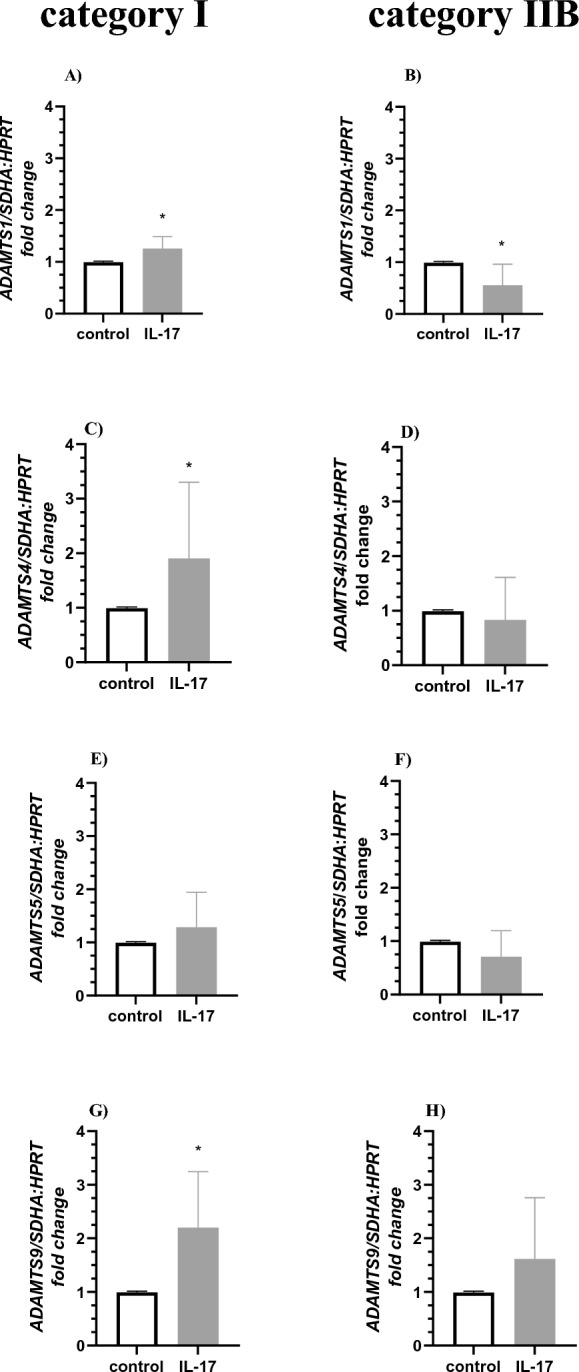
The effect of IL-17 on expression of *ADAMTS* mRNA expression in equine endometrial fibroblasts. IL-17 treatment (10 ng/ml) on (**A**,**B**) *ADAMTS1*, (**C**,**D**) *ADAMTS4*, (**E**,**F**) *ADAMTS5*, (**G**,**H**) *ADAMTS9* mRNA transcription in in vitro cultured fibroblast derived from (**A**,**C**,**E**,**G**) categories I and (**B**,**D**,**F**,**H**) IIB *endometria*. Results are presented as a fold change. Asterisks denote statistical differences (*P < 0.05) as determined by the nonparametric Mann–Whitney *U* test.

Interleukin 17 treatment increased *ADAMTS1*, *ADAMTS4,* and *ADAMTS9* mRNA transcription in cultured fibroblasts derived from category I *endometrium* (Fig. 7A,C,G; P < 0.05). However, IL-17 treatment decreased *ADAMTS1* mRNA transcription in cultured fibroblasts derived from category IIB *endometrium* (Fig. 7B; P < 0.05). Interleukin 17 treatment did not affect the expression of *ADAMTS4*, *ADAMTS5, ADAMTS9* in fibroblasts derived from category IIB (Fig. 7D,F,H; P > 0.05) and the expression of *ADAMTS5* in fibroblasts derived from category I *endometria* (Fig. 7E > 0.05).

## Discussion

In the present study, the identified transcriptomic changes associated with endometrosis suggest that inflammation and metabolic changes are features of mild and moderate stages of endometrosis. In addition, the results of this study suggest that cytokines secreted, possibly by macrophages and T helper cells affect the expression of genes associated with ECM remodeling.

### Fibrosis development and progression

The analysis of selected molecular and cellular functions and canonical pathways predicted to be associated with DEGs in *endometria* at different stages of endometrosis reflect events in the development and progression of tissue fibrosis described in other tissue.

In the present study, in the mild (IIA) and moderate (IIB) categories vs category I *endometrium*, DEGs were annotated to inflammation of organs (e.g. *CXCR4*, *CD68*, *CD36*, *F8*, *NLRP3*, *TLR7*), cellular infiltration by leukocytes, macrophages, and phagocytes as well as cell movement of phagocytes and leukocytes (e.g. *ALOX5*), and cytokine quantity (e.g. *ALOX5*, *CD36*, *IL12RB2*, *NLRP3, PLD4, RNF128*). The importance of macrophages in fibrosis development is well established^16, 17^. In our study, an increase in the expression of *CD36, CD68,* and *CXCR4* in category IIA vs I *endometria* possibly points towards the role of macrophages in endometrosis development. Macrophages are known to drive fibrosis by producing profibrotic mediators including TGF-β1 and platelet-derived growth factor (PDGF) that directly activate fibroblasts and control extracellular matrix turnover by regulating the balance of MMPs and TIMPs^16^. The expression of CD68 was increased in rat models of liver fibrosis^18^ and in patients with intermediate and advanced stages of oral submucous fibrosis^19^ and idiopathic pulmonary fibrosis (IPF)^20^. The infiltration of macrophages (CD68^+^ cells) in category I and IIA *endometria* using immunofluorescence staining was confirmed. These results suggest the potential role of macrophages in the development of endometrosis in mares. However, the results from the transcriptomic analysis are not clear which population of macrophages is predominant in the endometrial tissue at different stages of endometrosis.

Chemokine CXCR4, a transmembrane protein receptor, expressed by most cell types, including macrophages is involved in processes such as cell proliferation and tissue regeneration^21^. Results of many studies revealed dysregulated CXCR4 expression in fibrotic tissue. An increased number of CXCR4^+^ cells were observed in the lung tissue of patients with IPF^22^ and, recently, it was suggested as a biomarker for IPF in humans^23^. Additionally, CXCR4 expression increased after renal injury and its sustained activation enhanced the fibrotic response^24^.

In a more advanced stage of endometrosis, in category IIB vs I, DEGs were annotated to the pulmonary fibrosis idiopathic signaling pathway (e.g. *ACTA2*, *AREG*, *ADAMTS1*, *WNT4*, *WNT11*), growth of connective tissue (e.g.* AGER*, *TIMP1*) and cell proliferation of fibroblasts (e.g.* AGER*, *FADD*, *FTH1*, *MED31*, *NUPR1*, *PDGFD*, *SERPINE1*, *TGFB1I1*). Moreover, our data showed the changes in the expression of genes annotated to apoptosis (e.g. *AAMDC*, *AATK*, *ACER2*, *AGER*, *CASP2*, *NUPR1*), necrosis (e.g.* AGER*, *ATF3*) and cell homeostasis (e.g. *AGER*, *AQP1*, *ATP13A4*, *HK2*, *HKDC1*). According to our results, *NUPR1*, *ATF3 AGER*, and *CASP2*, which are positive regulators of apoptotic processes, were up-regulated in category IIB endometria. Though the role of apoptosis in tissue fibrosis is not very well studied, apoptosis was shown to be related to the cell type in fibrotic tissue^25–27^. It was demonstrated that increased apoptosis of alveolar epithelial cells and decreased apoptosis of fibroblasts may play an important role in the pathogenesis of lung fibrosis^25^. Increased expression of apoptotic factors, NUPR1 and ATF3 has been reported to activate renal fibrosis in rats^26^ and liver fibrosis in humans and mice^27^, respectively. However, it has to be underlined that apoptotic factors were found to have roles different than only induction of apoptosis and their role need to be investigated in endometrosis.

Additionally, the results of our study demonstrated the changes in the expression of genes associated with mitochondrial dysfunction (e.g.* COX17*, *COX6B1*, *CPT1B*, *FIS1*, *GLRX2*) and oxidative phosphorylation (e.g.* MT-ATP6*, *MT-CO1*, *MT-CO2*, *NDUFA3*, *NDUFS6*, *UQCRB*) in category IIB endometria. There is a growing body of evidence that mitochondrial dysfunction contributes to the development and progression of fibrosis^28^. Results of our study demonstrated alteration in the expression of genes connected to the respiratory chain *(COX6B1)*, proton transmembrane transporter activity (*MT-ATP6, MT-CO1)* mitochondria fission (*FIS1*), and redox signal transduction (*GLRX2*) in category IIB endometria. According to other study, the expression of COX17 was up-regulated in ischemia–reperfusion injury AKI mice which suggests that COX17 plays a role in the development of renal fibrosis^29^. Furthermore, Zhou et al.^30^ showed that the protein abundance of FIS1, involved in the fragmentation of the mitochondrial network and its perinuclear clustering, was up-regulated in the human fibrotic liver which suggests that FIS1 can promote HSC activation.

### Metabolic changes

The presented study points to potential novel factors and pathways which has to be taken into consideration as important players in the development of fibrosis. Cellular metabolism is a critical regulator of changes in cellular function. The altered metabolism of carbohydrates, lipids, proteins, and hormones has been documented in lung, liver, and kidney fibrosis^31, 32^. The changes in cellular metabolism described in other fibrotic tissue have not been pointed out so far in equine endometrosis. In our study, the possible metabolic changes, especially in category IIA vs I endometria, were connected to metabolism and molecular transport of lipids (e.g.* ABHD3*, *ALOX5*, *FABP5*, *KDSR*, *PLPP6*), carbohydrates (e.g. *ALDOB*, *FABP5*, *FOXO1*, *FUT2*, *HK2*), and amino acids (e.g. *SLC1A1*, *SLC25A29*, *SLC38A9*, *SLC6A20*, *SLC7A2*). Results of our research showed that *HK2* mRNA transcription declined in all stages of endometrosis. Accumulating evidence points to the role of HK2 in fibrosis development. The role of HK2 in fibrosis seems to be dependent on the affected organ. In the heart, Wu et al.^33^ demonstrated that a reduction in HK2 levels caused altered remodeling of the heart in ischemia/reperfusion by increasing cell death, fibrosis and reducing angiogenesis. The knockdown of HK2 resulted in exaggerated cardiac hypertrophy via increased reactive oxygen species production^34^. However, Yin et al.^35^ indicated that HK2 coupled glycolysis with the profibrotic actions of TGF-β in pulmonary fibrosis. In equine fibroblasts derived from category I and IIA endometria, TGF-β1 up-regulated mRNA transcription of *HK2* and down-regulated it in fibroblasts derived from category IIB endometria. Probably, the level of HK2 is down-regulated in the course of fibrosis, but at the beginning of its development, TGF-β1 stimulates its transcription.

### Inflammatory mediators and their effect on tissue remodeling

Our results link fibrosis to the inflammatory response which can be a result of cellular infiltration by macrophages, Th1 and Th2 activation pathways (e.g.* CXCR4*, *IL12RB2*), IL-13 (e.g.* AGR2*, *CD36*, *DEFB1*, *SPDEF*), IL-17 signaling pathways (e.g.* CXCL1*, *DEFB1*, *LIF*, *MMP9*, *RASD1*), and fMLP signaling in neutrophils (e.g.* RASD1*). Moreover, a huge number of inflammatory mediators such as TGF-β1, IL-4, IL-13, and IL-17 were determined as regulators of DEGs. Immune cells play an important role in the development of fibrosis by their effect on surrounding cells through secreted cytokines^36^. As suggested previously, the profibrotic action of TGF-β1, IL-1β, and IL-6 in the development of mare endometrial fibrosis can be connected to myofibroblast differentiation, fibroblast proliferation, MMP expression, and gelatinolytic activation, as well as synthesis of ECM^10–13, 37^.

In the current study, we were interested in the action of TGF-β1, which is the most potent profibrotic growth factor, on mRNA transcription of *ADAMTS* family members in endometrial fibroblasts. In general, ADAMTS are multi-domain matrix-associated zinc metalloendopeptidases that play multiple roles in tissue morphogenesis and pathophysiological remodeling, inflammation, and vascular biology^38^. These protein family members are strongly related to the TGF-β signaling and are involved in the ECM remodeling process^39, 40^. Recent studies suggest the role of ADAMTS members in the development of fibrosis as the important players in the turnover of ECM in various tissues and their altered regulation has been implicated in diseases such as cancer, osteoarthritis, and cardiovascular disease^41–44^. Results of our study showed that the expression of *ADAMTS1*, *4*, and *5* were up-regulated, while *ADAMTS9*, *12,* and *19* were down-regulated in endometrial tissue in category IIB. It suggests their important role in the development of endometrosis. Although the biological functions of the majority of the distinct ADAMTS subtypes remain poorly characterized, there is a growing interest in their role in the pathogenesis of tissue fibrosis. Dysregulated expression of members of ADAMTS family genes/proteins was found to be associated with fibrosis in the kidney and the lung^42, 43, 45, 46^. The expression and activation of ADAMTS-4 are augmented by several molecules, such as IL-1, tumor necrosis factor (TNF), TGF-β1, IL-17 as well as fibronectin^44^. These suggest their important function in endometrial fibrotic processes in mares. Thus, we further explored the effect of select upstream regulators on ADAMTS genes in equine endometrial fibroblasts derived from different stages of endometrosis. The effect of TGF-β1 on *ADAMTS1* and -*9* mRNA transcription differed in equine endometrial fibroblasts depending on endometrosis stage. Moreover, TGF-β1 treatment increased *ADAMTS4* but decreased *ADAMTS5* mRNA transcription in endometrial fibroblast.

We also determined the effect of IL-4, IL-13, and IL-17 on *ADAMTS* mRNA transcription in equine endometrial fibroblasts. The knowledge about the role of these cytokines in the development of endometrosis in mare remains unclear. However, in recent years, the role of T helper cells in the development of fibrosis in different organs has been  intensively investigated^47^. Results of the study showed that DEGs were annotated to Th1 and Th2 activation pathways as well as IL-13 and IL-17 signaling pathways. In general, it was demonstrated that IL-17 exhibits both profibrotic and antifibrotic functions in different organs in a disease-specific manner. The cytokines produced by Th2 (IL-4 and IL-13) act as a profibrotic factor in skin fibrosis^48^. However, to the best of our knowledge, the effect of IL-4, IL-13, and IL-17 on the mRNA transcription of *ADAMTS* family members in fibroblasts has not studied so far. Their importance in fibrosis can be attributed to a narrow substrate specificity that makes them potentially better pharmaceutical targets.

## Conclusion

The changes in gene expression appear to reflect the changes occurring in *endometria* at various stages of endometrosis. However, our results only suggest a possible mechanism of action and these findings need to be investigated by designing functional studies. In the mild stage of endometrosis compared to category I *endometria*, DEGs are annotated to inflammation, abnormal metabolism, wound healing, and quantity of connective tissue. In the moderate stage of endometrosis compared to category I, DEGs are annotated among others to inflammation, fibrosis, cell death, cellular homeostasis, and mitochondrial dysfunction. The changes of *ADAMTS-1*, *-4*, *-5*, *-9*, in fibrotic *endometrium* as well as in endometrial fibroblast in response to TGF-β1, IL-4, IL-13, and IL-17 suggest the important role of these factors in the development of endometrosis.

## Material and methods

Uteri used in the experiments (42 in total; 27 in experiment 1 and 15 in experiment 2) were obtained post-mortem from cyclic mares at a local abattoir (Rawicz, Poland). Procedures were reviewed and accepted by the Local Ethics Committee for Experiments on Animals in Olsztyn, Poland (Approval No. 51/2011). The collection of material was conducted between April and June. Normally cycling cold-blooded mares (weighing from 400 to 600 kg) at age from 2 to 20 were used in this study. The mares were clinically healthy, as declared by official government veterinary inspection and individual veterinary histories of animal health. Immediately before death, peripheral blood samples were collected into heparinized tubes (Monovettes-Sarstedt, Numbrecht, Germany). The material was collected within 5 min after slaughter. The follicular phases of the estrous cycle were identified based on progesterone (P_4_) analysis of blood plasma and the macroscopic observation of ovaries. The follicular phase was characterized by the absence of an active corpus luteum (CL) and the presence of follicles of various sizes, but always at least one follicle > 35 mm in diameter, with a concentration of P_4_ < 1 ng/ml as described previously^9^. All *endometria* were confirmed to be free from inflammation, by microscopic evaluation of endometrial smears collected with sterile swabs before the *endometrium* was excised from the myometrium and stained with Diff-Quick^49^. The presence of bacteria detected by cytological examination was the criterion for the exclusion of tissue samples from the experiments. The presence of more than two neutrophils per four microscopic fields (mag = 400X) indicates acute endometritis^50^.

Endometrial tissue was divided into two parts, one part was placed in 4% paraformaldehyde for histological analysis after hematoxylin–eosin staining and the other part was stored in RNAlater (#AM7021; Invitrogen, Burlington, ON, Canada) for RNA-seq. The horns of uteri (n = 15) for cell culture were kept in cold sterile physiological saline with 0.01% of antibiotic antimycotic (AA) solution containing penicillin, streptomycin, amphotericin (A5955, Sigma-Aldrich) and transported to the laboratory on ice. After hematoxylin–eosin staining, *endometria* were retrospectively assigned to categories I, IIA, or IIB according to the Kenney and Doig classification^2^.

### The isolation and culture of fibroblasts

The fibroblasts were isolated from mare *endometrium*, cultured, and passaged, as previously described with modifications^10^. Briefly, the uterine lumen was washed three times with 10 ml of sterile Hanks’ balanced salts (HBSS; H1387; Sigma-Aldrich) containing 0.01% of AA solution. A uterine horn was slit open with scissors to expose the endometrial surface. Endometrial strips were excised from the myometrium layer with a scalpel, washed once with sterile HBSS containing 0.01% of AA solution, and cut into very small pieces (1–3 mm^3^) with a scalpel. The minced tissues were digested once by stirring for 45 min in 100 ml of sterile HBSS containing 0.05%, (w/v) collagenase I (C2674, Sigma-Aldrich), 0.005% (w/v) DNase I (11,284,932,001; Roche), 0.01% AA, and 0.1% (w/v) bovine serum albumin (BSA; A9418, Sigma-Aldrich). Then, the cell suspension was filtered through the 100 μm, 70 μm, and 40 µm strainers to remove undigested tissue fragments and centrifuged at 100×*g* for 10 min. To lyse red blood cells, the cell pellet was resuspended and gently mixed with 1 ml of Red Blood Cell Lysing Buffer Hybri-Max™ (R7757; Sigma-Aldrich), then washed three times by centrifugation (4 °C, 100×*g*, 10 min) in HBSS supplemented with antibiotics and 0.1% (w/v) BSA. The final pellet of endometrial cells was resuspended in FBM™ Basal Medium (CC-3131, LONZA) supplemented with FGM™-2 SingleQuots™ supplements. The cells were counted using a Countess™ 3 FL Automated Cell Counter. The viability of endometrial cells was higher than 95% as assessed by the trypan blue exclusion test.

The dispersed cells were seeded separately at a density of 5 × 10^5^ viable cells/ml and cultured at 38.0 °C in a humidified atmosphere of 5% CO_2_ in the air. To purify the fibroblast population, the medium was changed 18 h after plating, by which time selective attachment of fibroblasts had occurred, and other types of endometrial cells were eliminated (i.e. epithelial and endothelial cells). The medium was changed every second day until the cells reached confluence. The fibroblast homogeneity was confirmed using immunofluorescent staining for vimentin based on the protocol described recently [^51^; data not shown]. The purity of fibroblast after isolation was around 96%. After reaching 90% of confluency, the cells were cryopreserved as described previously^52^.

### Experiment 1. Transcriptomic analysis of endometrial tissue at different stages of endometrosis at the follicular phase of the estrous cycle

#### RNA extraction, purification, and degradation analysis

The endometrial tissues were divided into three groups (n = 9 for each) according to Kenney and Doig’s categories: I, IIA, and IIB^2^. Total RNA was isolated from around 60 mg of endometrial tissue and homogenized with the use of TriReagent® (T9424; Sigma-Aldrich) and controlled in terms of quality using the Agilent TapeStation2200 system. Within each group, every three RNA isolates were pooled in equimolar ratios into one sample to obtain three replicates (pools). The pooling of samples was performed to reduce the experimental costs and the variability among individual samples and at the same time have more individuals within an experiment.

RNA integrity number (RIN) was assessed for each RNA isolate using Agilent 2100 system and Expert software (Agilent Technologies, Inc., Santa Clara, CA, USA). Only samples with a RIN above 8.0 were processed further. In total, 500 ng of RNA was used for library construction with the TruSeq RNA Sample Prep v2 kit (Illumina, San Diego, CA).

#### Library preparation

Standard library preparation steps including mRNA selection, fragmentation, cDNA synthesis, end repair, adenylation, indexed adapters ligation, and amplification were followed by a qualitative evaluation (Agilent TapeStation 2200) and quantitation (Qubit, Thermo Fisher Scientific). The libraries were eventually sequenced in a single 50 bp run (1 × 50 bp) on the HiScanSQ system using the TruSeq SBSv3 Sequencing kit, to obtain 15–20 million reads/sample.

#### Data analysis

Raw reads were controlled for quality using FastQC software and filtered with Flexbar software to remove adapters and those with a Phred quality score below 20 and read length below 30 nt. Filtered reads were mapped against *Equus caballus* reference genome assembly (EquCab3) with a TopHat2 software set to single-end reads and the “bowtie2 sensitive” option. The reads mapped to individual genes were counted using HTSeq software with ‘Union’ mode and GTF annotation file (Ensembl annotation version 104). Differential expression analysis was performed with DESeq2 software in pairwise comparisons. The quality of RNA-Seq read mappings was controlled using RseQC software. Only genes with adjusted P-value (after FDR correction using the Benjamini–Hochberg procedure, q-value) < 0.05 were considered as differentially expressed (DE). The characteristics of a given RNA-seq data set among the groups were evaluated using principal component analysis (PCA) (Supplementary Data 5).

To determine the gene network, the obtained data were analyzed using Ingenuity Pathways Analysis (IPA) tools (Ingenuity Systems, Mountain View, CA), a web-delivered application that enables the identification, visualization, and exploration of molecular interaction networks in gene expression data.

Raw sequencing reads obtained from all analyzed samples were deposited in the publicly available SRA (Sequence Read Archive) NCBI database under accession number PRJNA880454.

### Experiment 2. The effect of cytokines on the differentially expressed genes in endometrial fibroblasts

In Experiment 1, DEGs in categories IIA vs I and IIB vs I *endometria* were observed to be regulated by TGF-β1, IL-4, IL-13, and IL-17. Thus, in Experiment 2, we aimed to determine how chosen cytokines (TGF-β1, IL-4, IL-13, IL-17) affect the expression of selected DEGs connected to extracellular matrix remodeling (ADAMTS family members) and cell metabolism (*HK*). In addition, using endometrial fibroblasts isolated from different endometrosis categories, we evaluated whether the response of fibroblasts to a given factor differs depending on the stage of endometrosis.

For in vitro experiments, thawed fibroblasts isolated previously from mare *endometria* (n = 5) were seeded on T75 cm^2^ in FBM™ Basal Medium supplemented with FGM™-2 SingleQuots™ supplements and ascorbic acid (100 ng/ml; A4544; Sigma-Aldrich). After reaching 90% confluence, fibroblasts were trypsinized and seeded on 24-well plates. When fibroblasts from passage 1 reached desired 80% confluence, the culture medium was replaced with starvation medium: Dulbecco’s Modified Eagle’s Medium/Nutrient Mixture F-12 Ham (DMEM/Ham’s F-12; D2906; Sigma-Aldrich) supplemented with 0.01% of AA solution, ascorbic acid (100 ng/ml) and 0.1% (w/v) BSA, and the cells were incubated at 38.0 °C in 5% CO_2_. After the starvation, cells were treated with TGF-β1 (10 ng/ml; 100–21; human recombinant; PeproTech), IL-4 (10 ng/ml; RP0003E, equine recombinant, Kingfisher Biotech), IL-13 (10 ng/ml; RP0102E, equine recombinant, Kingfisher Biotech) or IL-17A (10 ng/ml; RP0078E, equine recombinant, Kingfisher Biotech) for 48 h. All doses of cytokine were selected based on previous studies: TGF-β1^10^, IL-4^53^, IL-13^54^, and IL-17^55^. After 48 h of the treatments, the cells were dispersed with 1 mL TRI Reagent® and stored at − 80 °C for subsequent RNA extraction and qPCR.

#### RNA extraction and cDNA synthesis

Total RNA was extracted using TRI Reagent® according to the manufacturer’s instructions. The concentration and quality of RNA were determined spectrophotometrically and by agarose gel electrophoresis. The ratio of absorbance at 260 and 280 nm (A260/280) was approximately 1.8–2. The RNA samples were kept at − 80 °C. Dnase I (AMPD-1; Sigma-Aldrich) was used according to the manufacturer’s directions for eliminating DNA from RNA samples before qPCR. Total RNA (1 μg) was reverse transcribed using a ThermoScript RT-PCR System according to the manufacturer’s directions (no. 11146-016; Invitrogen). The cDNA was stored at − 20 °C until qPCR was carried out.

#### qPCR

To validate the RNA-Seq results, the expression level of selected DEGs was confirmed by qPCR. The same samples were used for both, RNA-Seq and qPCR analysis. However, to validate the results of RNA-seq the samples were not pooled but all individual samples were used. Additionally, the qPCR was used to determine the effect of selected cytokines on gene expression in endometrial cells in vitro. qPCR was performed in a 7900HT Fast Real-Time PCR System using TaqMan Universal Master Mix II (4,440,049; Applied Biosystems, Foster City, CA, USA) with 384-well plates. All samples were run in duplicates. For measurements of mRNA transcription of *ADAMTS1* (cat. no. Ec03469970_m1), *ADAMTS4* (cat. no. Ec03469176_m1), *ADAMTS5* (cat.no. Ec03470669_m1), *ADAMTS9* (cat.no. Ec06952347_m1), *CXCR4* (cat. no. Ec07070263_s1), *HK2* (cat.no. Ec03467576_m1), *SLC25A29* (cat. no. Ec06979775_m1), *HPRT* (cat. no. Ec03470217_m1), *SDHA* (cat. no. Ec03470487_m1), *GAPDH* (cat. no. Ec03210916_gH), *B2M* (cat. no. Ec03468699_m1), *RPL32* (cat. no. Ec06951800_m1), *RPS18* (cat.no. Ec06969343_g1), *RPS28* (cat. no. Ec06997545_g1), Single Tube TaqMan Gene Expression Assays (Life Technologies Thermo Fisher Scientific) were used. The selection of the most adequate reference genes was done using NormFinder software^56^. As *SDHA* and *HPRT* were found to have the most stable expression across the endometrosis categories, gene expression data were normalized to the average geometric mean of these two genes.

The reaction mixture for the qPCR assay comprised 5 μL TaqMan Universal PCR Master Mix, 0.5 μL TaqMan probe, 3 μL DNA (10 ng), and 1.5 μL nuclease-free water for a final volume of 10 μL. As a negative control, nuclease-free water instead of template cDNA was used. cDNA amplification was performed under the following conditions: initial denaturation for 10 min at 95 °C, followed by 40 cycles of 15 s at 95 °C and 1 min at 60 °C. The data were analyzed using the method described previously^57^. The relative concentration of mRNA (R0) for each target and reference gene (*SDHA; HPRT*) was calculated using the equation R0 = 1/(1 + E)^Ct^_,_ where, E is the average gene efficiency and Ct is the cycle number at the threshold. The relative gene expression was calculated as R0_target gene_/R0_reference gene_ and was expressed in arbitrary units.

#### Immunofluorescence staining

The immunofluorescence staining was performed to determine the infiltration of macrophages in the categories I (n = 3) and IIA (n = 3) *endometria* to support results obtained from the transcriptomic analysis. The immunofluorescence staining was performed as described previously^51^. Briefly, formalin-fixed endometrial samples were processed, embedded in paraffin, and sectioned at 5 μm. Sections were deparaffinized (xylene I for 10 min, xylene II for 10 min) and rehydrated through a graded alcohol series (100% ethanol for 2 min, 96% ethanol for 2 min, and 70% ethanol for 2 min). Rehydration was followed by antigen retrieval in sodium citrate buffer (10 mM Sodium citrate, 0.05% Tween 20, pH = 6) at 95 °C for 25 min. Subsequently, the endometrial sections were permeabilized with PBS containing 0.3% TritonX-100 for 10 min, washed one time for 5 min in PBS, and blocked with 1% BSA and 5% normal goat serum in PBS for 1 h at room temperature. The endometrial sections were incubated overnight with CD68 + primary antibody (1:100, cat. no. 14–0681-82; Invitrogen) at 4 °C. Afterward, the sections were washed three times for 5 min with PBS and incubated with secondary antibodies labeled with Alexa Fluor 594 (1:400, cat no. A-11007, Thermo Fisher Scientific). The slides were washed three times for 5 min with PBS and mounted in Fluoroshield with DAPI (Sigma-Aldrich; F6057).

The number of CD68^+^ cells was counted manually in 15 regions that were randomly selected from 15 images at 20 × magnification from each endometrial section. The scoring results were shown as mean ± SD number of immunopositive cells per µm^2^ of the tissue section.

### Radioimmunoassay

Plasma concentrations of P_4_ were assayed by RIA (Diasource; KIP1458). All samples were run in duplicate. The P_4_ standard curve ranged from 0.12 to 36 ng/ml. The intra- and inter-assay CVs were on average 8% and 10%, respectively. The sensitivity of the assay was 0.05 ng/ml. The validation of RIA in horse blood plasma was done as described previously^58^.

### Statistical analysis

Data are shown as the mean ± SD. For each analysis, the Gaussian distribution of results was tested using the D'Agostino & Pearson normality test (GraphPad Software version 9; GraphPad, San Diego, CA). Whenever the assumptions of normal distribution were not met, nonparametric statistical analyses were done. In Experiments 1 and 2, the significant differences were determined by the nonparametric Mann–Whitney *U* test. The endometrial *HK2* mRNA transcription in categories I, IIA, and IIB was determined by using parametric one-way ANOVA followed by a Newman-Keuls comparison test. The results were considered significantly different when P < 0.05.

### Ethics approval and consent to participate

Procedures were reviewed and accepted by the Local Ethics Committee for Experiments on Animals in Olsztyn, Poland (Approval No. 51/2011). All methods were carried out in accordance with relevant guidelines and regulations. All methods are reported in accordance with ARRIVE guidelines for the reporting of animal experiments.

### Supplementary Information

Supplementary Table 1.

Supplementary Table 2.

Supplementary Table 3.

Supplementary Table 4.

Supplementary Information.

Supplementary Legends.

## Data Availability

The datasets used and/or analyzed during the current study are available from the corresponding author upon reasonable request. NGS results were deposed in the publicly available SRA (Sequence Read Archive) NCBI database under accession number PRJNA880454.
