# Supplementary material for: Transcriptomic profiling of mare endometrium at different stages of endometrosis
Source: Sci Rep. 2023 Sep 27;13:16263. doi: 10.1038/s41598-023-43359-5 (PMC10533846; doi:10.1038/s41598-023-43359-5)
Supplement: Supplementary file 1 — Supplementary Table 1. [file 41598_2023_43359_MOESM1_ESM.docx]

| Index. | Sample name | Raw reads | Filtered reads | Uniquely mapped reads | % Uniquely mapped reads |
| --- | --- | --- | --- | --- | --- |
| 1. | endo_cat_I_1 | 16 205 442 | 16 164 166 | 14 588 378 | 90.25 |
| 2. | endo_cat_I_2 | 17 457 005 | 17 410 119 | 14 234 903 | 81.76 |
| 3. | endo_cat_I_3 | 14 651 614 | 14 610 766 | 13 338 213 | 91.29 |
| 4. | endo_cat_IIA_1 | 16 496 028 | 16 462 438 | 14 816 444 | 90.00 |
| 5. | endo_cat_IIA_2 | 21 305 969 | 21 262 704 | 19 310 990 | 90.82 |
| 6. | endo_cat_IIA_3 | 18 352 813 | 18 228 482 | 16 506 783 | 90.53 |
| 7. | endo_cat_IIB_1 | 19 058 242 | 19 013 975 | 17 226 347 | 90.60 |
| 8. | endo_cat_IIB_2 | 18 350 010 | 18 309 249 | 15 877 137 | 86.72 |
| 9. | endo_cat_IIB_3 | 20 032 626 | 19 991 510 | 17 920 605 | 89.64 |
| mean | | 17 989 972 | 17 939 268 | 15 979 978 | 89.06 |

**Supplementary table 1. Statistical analysis of RNA sequencing reads in *endometrium* of mare at the follicular phase of the estrous cycle in category I, IIA and IIB endometria.**
