# Supplementary material for: Transcriptomic profiling of mare endometrium at different stages of endometrosis
Source: Sci Rep. 2023 Sep 27;13:16263. doi: 10.1038/s41598-023-43359-5 (PMC10533846; doi:10.1038/s41598-023-43359-5)
Supplement: Supplementary file 4 — Supplementary Table 4. [file 41598_2023_43359_MOESM4_ESM.docx]

**Supplementary table 4.** Results of RNA-seq experiment validation with qPCR. Category IIA, IIB, III vs I endometria.

| Gene symbol | qPCR | | RNA-seq | |
| --- | --- | --- | --- | --- |
|  | Log2fold | p-value | Log2fold | p-value |
|  | **Category IIA vs. I** | | | |
| **HK2** | -5.507 | 0.0051 | -4.105 | 2.20E-11 |
| **CXCR4** | 1.612 | 0.0070 | 1.230 | 0.00014 |
|  | **Category IIB vs. I** | | | |
| **ADAMTS9** | -2.0231 | 0.0238 | -1.787 | 0.00021 |
| **HK2** | -5.680 | 0.0159 | -2.508 | 0.00048 |
