# Supplementary figures and images for: Transcriptomic profiling of mare endometrium at different stages of endometrosis
Source: Sci Rep. 2023 Sep 27;13:16263. doi: 10.1038/s41598-023-43359-5 (PMC10533846; doi:10.1038/s41598-023-43359-5)

## Supplementary data 5

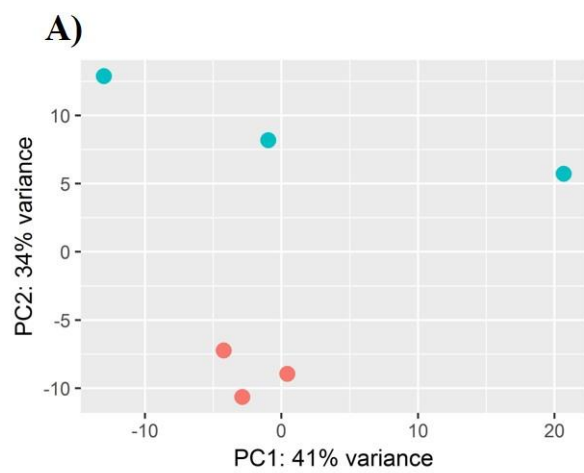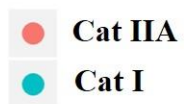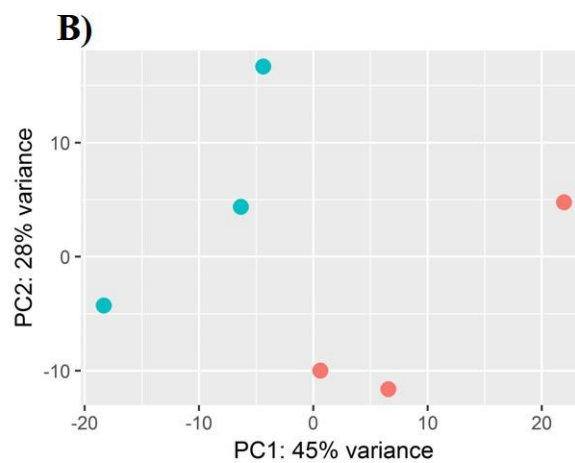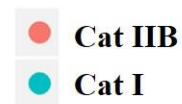

Supplement: Supplementary file 5 — Supplementary Information. [file 41598_2023_43359_MOESM5_ESM.pdf]
