# Supplementary material for: Transcriptomic profiling of mare endometrium at different stages of endometrosis
Source: Sci Rep. 2023 Sep 27;13:16263. doi: 10.1038/s41598-023-43359-5 (PMC10533846; doi:10.1038/s41598-023-43359-5)
Supplement: Supplementary file 6 — Supplementary Legends. [file 41598_2023_43359_MOESM6_ESM.docx]

**Supplementary data list**

**Supplementary table 1.** Statistical analysis of RNA sequencing reads in *endometrium* of mare at the follicular phase of the estrous cycle in category I, IIA and IIB endometria.

**Supplementary table 2.** A list of differentially expressed genes (DEG) in categories: IIA and IIB, *vs*I, as well as IIB vs IIA endometria.

**Supplementary table 3.** A list of comparison analyses, comparing disease and biological functions, canonical pathways, and upstream regulators of DEGs identified in categories IIA, IIB vs category I endometria and category IIB vs IIA. The analysis was done using ingenuity pathway analysis (IPA). **CP –** canonical pathways**, D&F –** disease and function**, UR -** upstream regulators.

**Supplementary table 4.** Results of RNA-seq experiment validation with qPCR. Category IIA vs category I endometria; category IIB vs category I *endometrium*.

**Supplementary data 5.** Principal component analysis (PCA) of RNA-seq data set in *endometrium* between categories: (A) IIA vs I; (B) IIB vs I.
